# Micro- and nano-Illite to improve strength of untreated-soil as a nano soil-improvement (NSI) technique

**DOI:** 10.1038/s41598-024-61812-x

**Published:** 2024-05-13

**Authors:** Mohadeseh Cheraghalikhani, Hamed Niroumand, Lech Balachowski

**Affiliations:** 1 Department of Civil Engineering, Faculty of Engineering, Buein Zahra Technical University, Qazvin, Iran; 2 Department of Geotechnical and Hydraulic Engineering, Faculty of Civil and Environmental Engineering, Gdansk University of Technology, Gdańsk, Poland

**Keywords:** Micro-Illite, Nano-Illite, Nano Soil-Improvement (NSI), Nano-cementation, Nano Ground Improvement (NGI), Engineering, Nanoscience and technology

## Abstract

Soil stabilization is a technique of improving the geotechnical properties of soils for various engineering applications. However, conventional stabilizers such as cement and lime have some limitations, such as high cost, environmental impact, and durability issues. Therefore, there is a need for alternative and innovative stabilizers that can overcome these challenges. This study introduces nano-Illite, a type of clay mineral, as a novel and effective soil stabilizer. Nano-Illite can form nano-cementation (NC) in soil, which is a process of enhancing the durability of various building materials. NC is also known as nano soil-improvement (NSI), a technique that has been developed in recent years. Four formulations of micro- and nano-Illite with concentrations of 0, 1, 2, and 3% were separately added to soil samples. The unconfined compressive strength (UCS) and the secant modulus at 50% of peak stress (E50) of the treated samples were measured and compared with the untreated samples. The results showed that 3% nano-Illite increased the UCS of soil by more than 2.2 times and the E50 by more than 1.5 times after 7 days of curing. Micro-Illite also improved the UCS and E50 of soil, but to a lesser extent. X-ray fluorescence (XRF), scanning electron microscopy (SEM), and X-ray diffraction (XRD) analyses revealed the micro- and nano-structures of the soil specimens and the performance of Illite as a nano-additive. This research demonstrates the effectiveness of nano-Illite in soil improvement as a NSI technique, and its potential to replace or reduce the use of conventional stabilizers. This study also contributes to the understanding of the mechanisms and factors that influence the NC process in soil.

## Introduction

Untreated soils are types of loose, loosely compacted soils that can be unstable and can cause problems for building foundations. Construction on untreated soils can lead to serious consequences, such as cracks in building structures and foundations, also highlighting studies that identify zones of soil vulnerability characterized by lower soil strengths, Atterberg limits exceeding construction guidelines, and areas prone to sensitive soil conditions^1–3^. Common techniques for improving soils include deep soil mixing, stone columns, jet grouting, pre-loading and related methods, but these techniques may not be sufficient for some types of soils. Using additives can be an effective way to improve soils and make them more stable. Additives can include traditional stabilizers like lime, cement, fly-ash, fiber-reinforced cemented, coal wasted, and related additives^4–8^. A few researchers evaluated the role of novel stabilizers with low carbon footprint and how the current study fits into the broader context in soil stabilization^1–3,9–12^. Each of these materials has its own advantages and disadvantages, and it is important to study the differences between them to understand the best options for improving untreated soils^13^.

According to these conditions, researchers are finding new techniques in soil improvement. There are few studies where Illite showed its potential in increasing the strength of soils^14–25^. Illite is one of recent environment friendly additives used to improve the geotechnical properties of soils although strength is limited. Nano-additive (NA) is one of the recent methods used to improve the geotechnical properties of soils. NA is a type of nano soil-improvement (NSI) techniques. Recently, nano-additives (NA) with outstanding mechanical properties has been used for soil stabilization in a few studies^4–8,14–25^. According to a literature review, no research has been conducted on the preparation of nanoparticles without the addition of chemicals using lower cost and energy by mechanical methods. The most studies have been used powder particles in soil stabilization^4–8,14–25^.

Most researchers and scientists have traditionally used micro and macro techniques for soil improvement. Although these techniques have enhanced soil properties, the role of nano techniques, or nano-micro methods, could potentially improve soil improvement techniques far more effectively than traditional methods. Nano Soil Improvement (NSI) has initiated a revolution in soil improvement, geotechnical engineering, and civil engineering because it can decrease void spaces in various cohesive and non-cohesive soils. NSI can be used in various applications such as ground improvement, foundation engineering, retaining walls, and off-shore systems.

The lack of research on the effects of the powder base of micro- and nano-additives on soils led this study to examine the effect of soil stabilization by micro-Illite and nano-Illite using the suspension method. Illite is a natural clay which is widely used in many fields, particularly in building and public works. It is used as an adjuvant in the manufacture of concrete, as a binder for soils, as a waterproofing agent for foundations, and as a containment material for nuclear waste. The advantages of Illite include its abundant availability, low cost, ability to retain water, and ability to improve the physical and chemical properties of materials to which it is added. This study was conducted to stabilize and improve the soil properties, considering the economic justification and optimal use of the Illite micro- and nano-particles, which had not been done in clayey sand soil previously.

Comparing the effects of micro-Illite and nano-Illite is an important research topic to understand the potential advantages of nano-Illite over micro-Illite. Nano-Illite may have unique properties that may make it more effective at improving clayey sand soils than micro-Illite but understanding the differences between the two is important to determining the best options for construction projects. This research aims to study the differences between micro-Illite and nano-Illite to improve clayey sand soils. The main objective of this research is to compare the effects of micro-Illite and nano-Illite with the addition of additives on clayey sand soils. This includes the study of the physical and mechanical properties of improved clayey sand soils, as well as their ability to withstand loads and maintain their stability over time. Tests will be carried out in the laboratory to understand the differences between micro-Illite and nano-Illite, on clayey sand soils.

Thus, in this research, a fixed amount of micro-Illite and nano-Illite added from lower to high concentrations at 0, 1, 2, and 3% was used as stabilizer to improve the geotechnical properties of clayey sand soils. All such clayey sand soils were evaluated to obtain Atterberg limits, elastic modules (E50), and unconfined compressive strength (UCS). All specimens were characterized to evaluate potential of micro- and nano-Illite in the enhancement of performance characteristics of clayey sand soils using SEM, XRD, and XRD analyses. The results of this research could be used to develop new techniques to improve untreated soils and to inform building standards and codes.

## Materials and methods

### Materials

**Figure 1:**
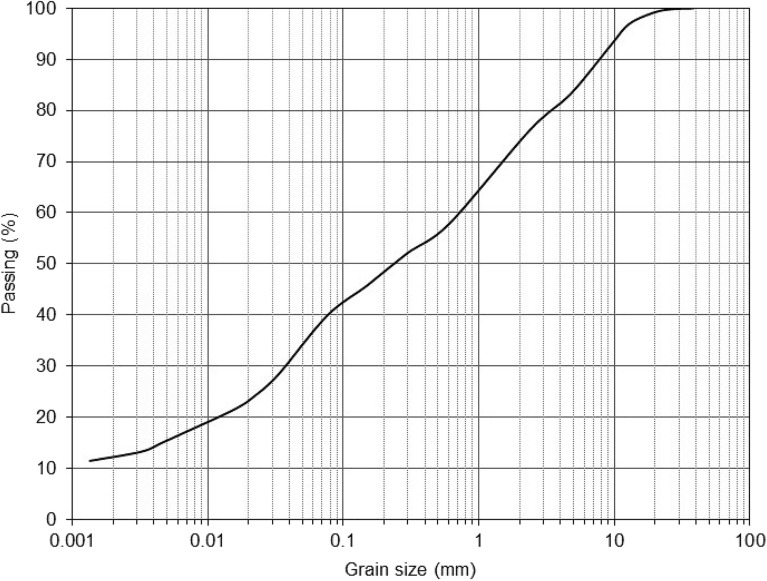
Grain-size distribution plot for the clayey sand soil.

Clayey sand soil samples were prepared from Survajin Aghigh in Qazvin Province, Iran. The sample soil was classified as clayey sand (SC) according to the unified soil classification system (USCS). The gradation curve, shown in Fig. 1, indicates that sample soil consists of 17% gravel, 43% sand, 40% clay and silt. Micro-Illite used in this research was bought in Tehran, Iran. The physicochemical of characteristics (XRF analysis) of micro-Illite used are given in Table 1.

**Table 1:** Chemical composition used micro-Illite (MI).

XRF-Illite	
Chemical compound (oxide)	Weight content (%)
Na_2_O	0.88
MgO	1.99
Al_2_O_3_	23.11
SiO_2_	53.77
Cl	0.62
K_2_O	6.21
CaO	1.59
TiO_2_	0.49
MnO	0.05
Fe_2_O_3_	2.99
BaO	0.17
L.O.I	8.13

### Nano-Illite production

The micro-Illite was used to produce nano-Illite in the present study. The Chemical composition of nano-Illite is given in Table 2. A ball mill used to produce nano-Illite powder. The nano-Illite’s powder was converted into suspension by a homogenizer mixer, termed as nano-Illite. Nano-Illite was stored at laboratory temperature to use.

**Table 2:** Chemical composition used nano-Illite (NI).

XRF-Nano Illite	
Chemical compound (oxide)	Weight content (%)
Na_2_O	0.88
MgO	2.00
Al_2_O_3_	23.19
SiO_2_	53.8
Cl	0.62
K_2_O	6.13
CaO	1.62
TiO_2_	0.45
MnO	0.05
Fe_2_O_3_	3.00
BaO	0.17
L.O.I	8.13

### Sample preparation

**Figure 2:**
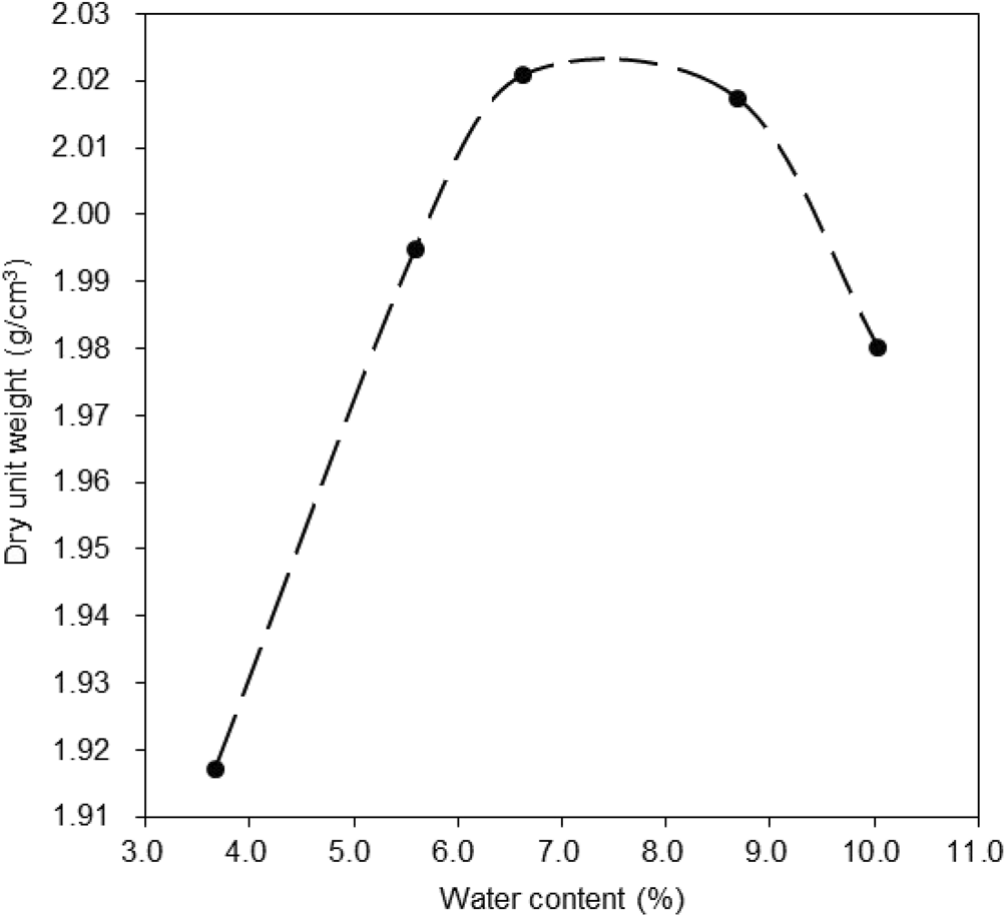
The optimum water content.

The clayey sand soil sample was dried in oven at 105 °C. Grain-size distribution plot for the clayey sand soil is given in Fig. 1. In this research, two type of additives in micro and nano size evaluated in soil mechanics laboratory. In the first type, micro-Illite with amount of 0, 1, 2, and 3% by dry weight of clayey sand soil sample were into the clayey sand soil. Clayey sand soil samples were mixed containing different concentrations of micro-Illite to obtain a homogenous mixture. In the second type, nano-Illite with amount of 0, 1, 2, and 3% by dry weight of clayey sand soil sample were into the clayey sand soil. Clayey sand soil samples were mixed containing different concentrations of nano-Illite to obtain a homogenous mixture. The final clayey sand soil specimens with 0, 1, 2, and 3% micro- and nano-Illite are coded as Illite and nano-Illite, respectively. The optimum water content to prepare soil samples was evaluated as per standard proctor compaction test shown in Fig. 2. All the specimens were tested in various curing times such as 1, 7, and 28 days.

The standard compaction test on the graph in Fig. 2 shows the maximum density of an untreated clayey sandy soil sample. The results of this test showed that the optimum water content of the clay sand sample has a direct impact on the dry unit weight. The standard compaction test of the clayey sandy sample shows that the compaction curve increases to 2.02 g/cm^3^ dry unit weight at 7.5% water content. The optimum water content of untreated clayey sandy is 7.5%, which corresponds to the maximum dry unit weight of 2.02 g/cm^3^.

### Atterberg limits

The Atterberg limits which determine the plasticity properties of a soil which are important for the valuation of the soil stability. The liquid limit, plastic limit, and plasticity index were tested. These values from clayey sandy soil specimens with micro- and nano-Illites were evaluated according to ASTM D 4318.

### Unconfined compressive strength (UCS)

A series of test specimens, each 50mm in diameter by 100mm in height, was prepared at an optimum moisture content for UCS determinations at various curing times. These values from clayey sand soil specimens with micro- and nano-Illites were evaluated based on ASTM D 2166. An axial strain rate of 1.27 mm/min was performed. These samples were tested for curing periods of 1d, 7d, and 28d. The secant modulus at 50% of peak stress (E50) of treated micro- and nano-samples in comparison to untreated specimen based on stress–strain behavior in UCS evaluated.

### XRF, XRD, and SEM analyses

X-Ray Fluorescence (XRF) analysis is an analytical technique that uses X-rays to excite atoms in a sample and measure the light emitted by the excited atoms. This light can be analyzed to determine the chemical composition of the sample. X-ray fluorescence analysis is a fast and accurate way to determine the composition of many types of materials, such as minerals, metal alloys, chemicals and consumer products. It is widely used in the mining, petroleum and metallurgical industries, as well as in environmental and occupational health and safety applications. These values from clayey sand soil specimens with micro- and nano-Illites were evaluated based on ASTM E1621-13 in XRF. X-ray diffraction (XRD) testing is a method used to study the crystal structure of a substance. It involves the use of X-rays to produce light diffraction on atoms in a crystal, producing an image that can be analyzed to determine the spatial arrangement of atoms in the crystal structure. The X-ray diffraction test made it possible to determine the crystal structure of Illite. XRD was evaluated to identify minerals and other crystal structure of soil samples including treated and untreated specimens with micro- and nano-Illites using X-ray diffractometer in Tehran, Iran. These values from clayey sand soil specimens with micro- and nano-Illites were evaluated based on BS EN 13,925–1 in XRD. Scanning Electron Microscopy (SEM) analysis is an imaging technique used to study the structure and composition of materials at the micro and nano scales. It involves using an electron beam to scan the surface of a sample and produce a two-dimensional image of its surface. The microstructures of all specimens with gold-coated were evaluated under a Scanning Electron Microscopy (SEM) in Tehran, Iran. These values from clayey sand soil specimens with micro- and nano-Illites were evaluated based on 5µ and 500 nm scales in SEM.

## Results and discussion

### Atterberg limits

**Figure 3:**
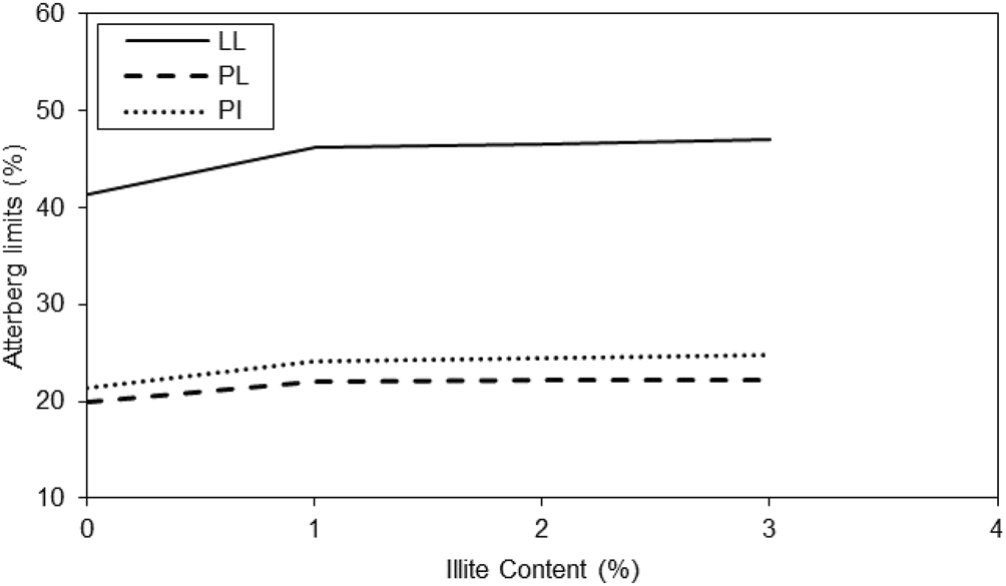
Variations of Atterberg limits of treated clayey sandy soil with various formulations of micro-Illite.

**Figure 4:**
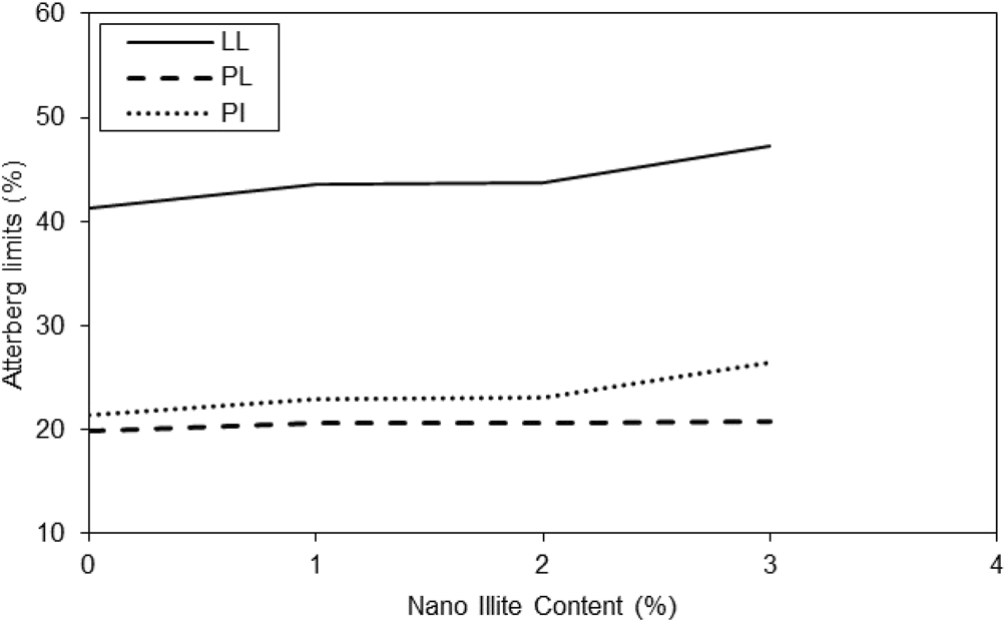
Variations of Atterberg limits of treated clayey sandy soil with various formulations of nano-Illite.

Generally untreated and treated clayey sandy soils in related to the role of water content for Atterberg limits were evaluated. According to Figs. 3 and 4, the liquid limit values of the clayey sandy specimens increased with an increasing amount of micro-Illite till 3% concentration and similar behaviors of plastic limit and plasticity index were observed. In nano-Illite, the liquid limit, plastic limit, and plasticity index of the treated specimens increased with an increasing amount of micro-Illite till 3% concentration were observed.

**Figure 5:**
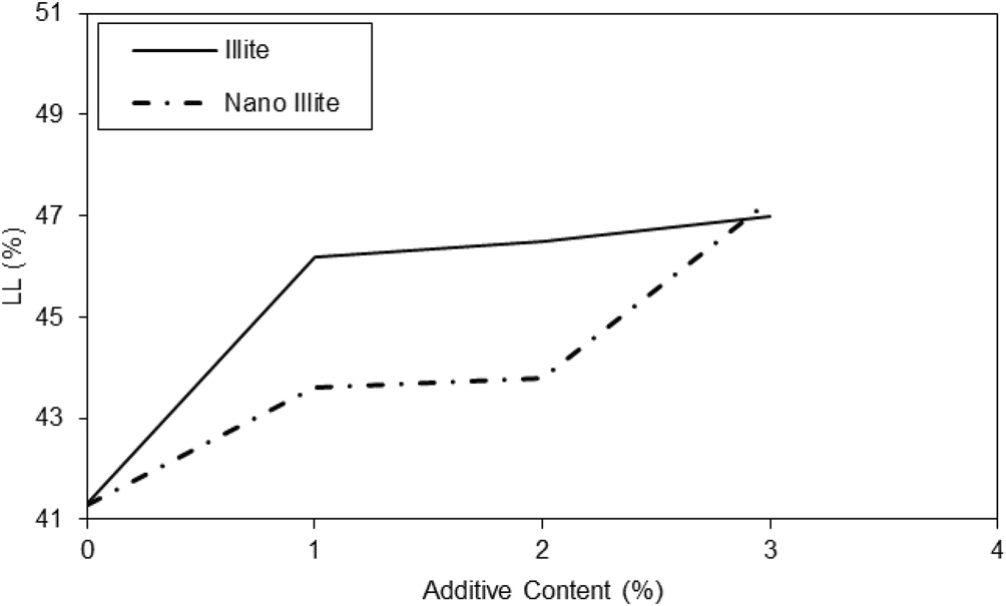
Variations of liquid limits of treated clayey sandy soil with various formulations of micro- and nano-Illite.

**Figure 6:**
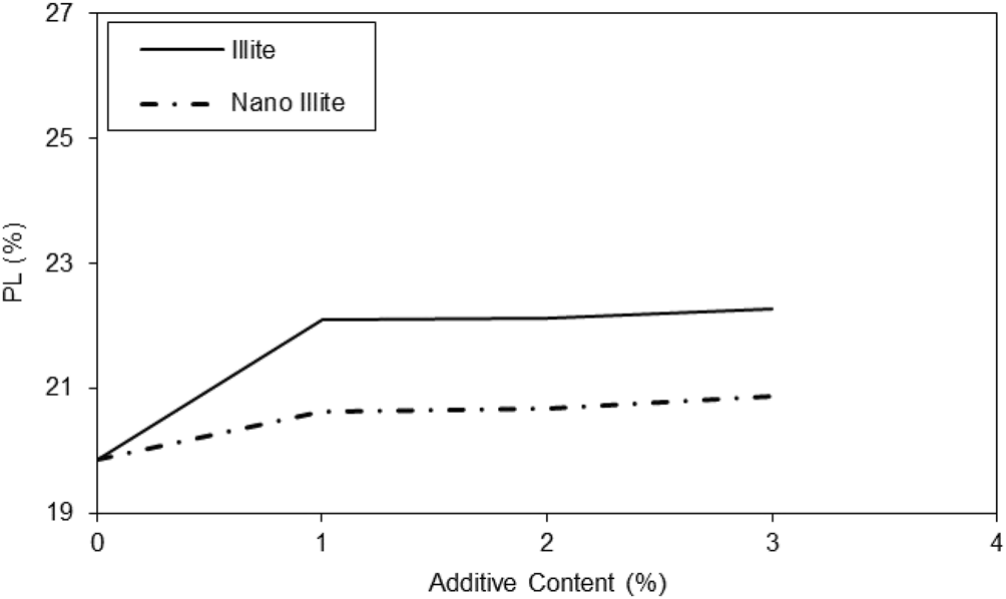
Variations of plastic limits of treated clayey sandy soil with various formulations of micro- and nano-Illite.

The results in the graph of Fig. 5 showed that the addition of additives had a significant effect on the liquid limit of micro-Illite and nano-Illite. In general, the addition of additives increased the liquid limit of micro-Illite but nano-Illite decreased the liquid limits. However, the effect was more marked for nano-Illite. It is observed that with a nano additive content of 1%, the liquid limit is 43% and with an additive content of 3%, the liquid limit reaches 47%. There is a notable increase in the liquid limit of 4% between the additive content of 1% and 3%. Increasing the additive content had a significant effect on the liquid limit of nano-Illite, significantly decreasing the liquid limit compared to micro-Illite. In general, the addition of additives increased the plastic limit of micro Illite. However, it has also been observed that the addition of additives can significantly increase the plastic limit of nano-Illite but it is lower than micro-Illite performance. Performance of various additive content are observed for the nano-Illite and the micro-Illite in Fig. 6. It can be assumed that with an additive content of 3%, the plastic limit of micro-Illite is the largest.

**Figure 7:**
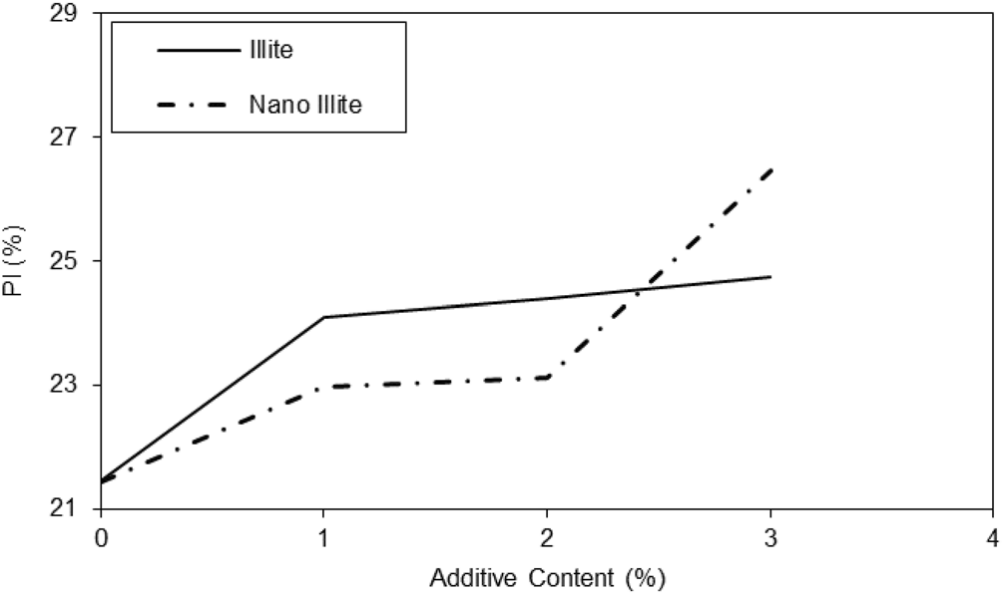
Variations of plasticity index of treated clayey sandy soil with various formulations of micro- and nano-Illite.

In the case of micro-Illite, the addition of additives increasingly increased the plasticity index, In the case of nano-Illite, the addition of additives increased the plasticity index, but this was done in several peaks, shown in Fig. 7. Once again, two notable peaks are observed at an additive content of 2% and 3%. With an additive content of 2%, the plasticity index of nano-Illite is lower than that of micro-Illite. While with a content of 3% the plasticity index of nano-Illite is higher than that of micro-Illite. It can also be assumed that it is with an additive content of 3% that the nano-Illite is the most the largest. It was observed an additive content of 4%, the plasticity index of nano-Illite is lower than that of micro-Illite. Addition of nano-Illite with nano-cementation aids flocculation and the clayey sandy particles that result in a better behavior rather than treated specimens with micro-Illite.

### Unconfined compressive strength (UCS)

**Figure 8:**
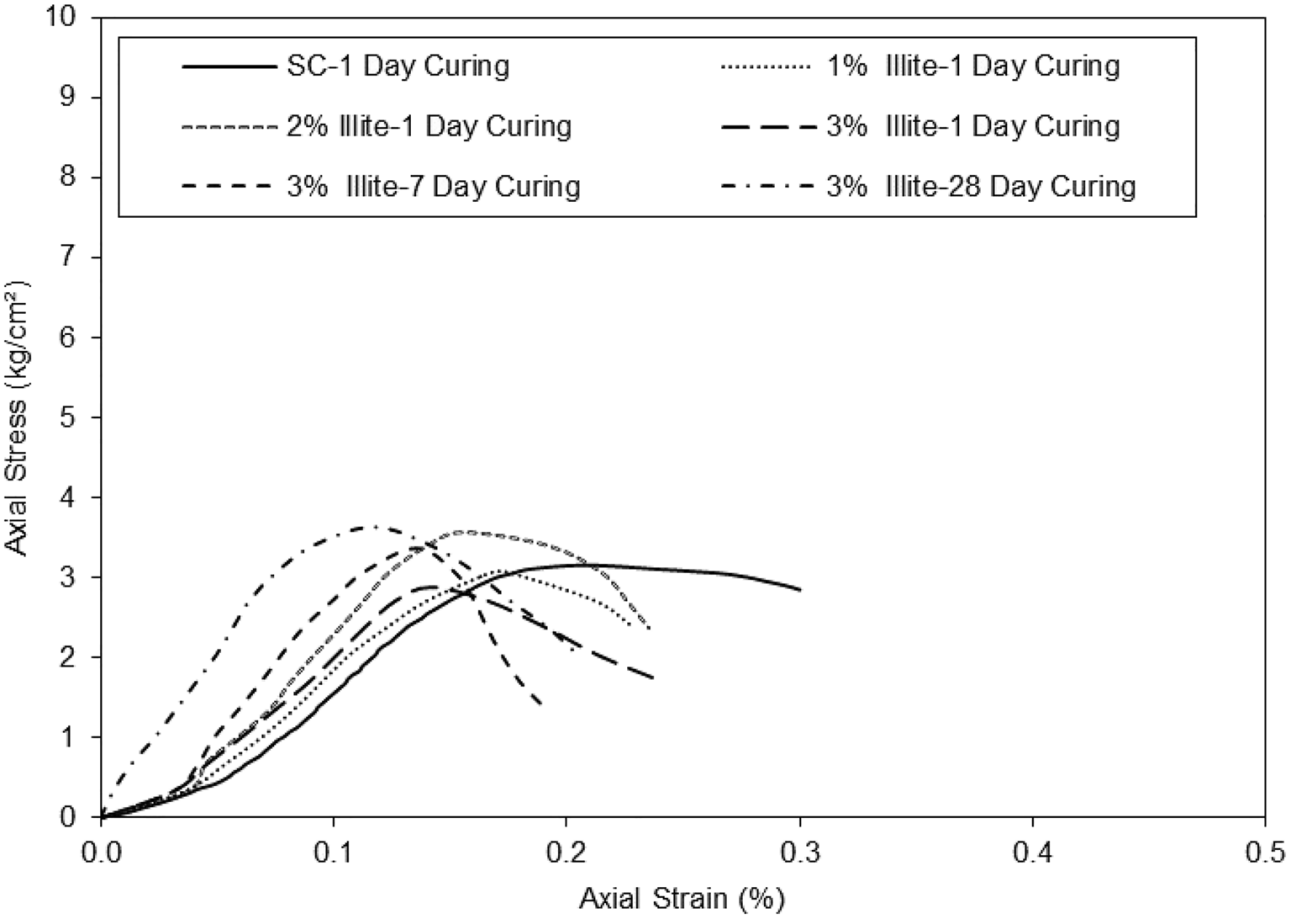
Stress shear strength versus strain for untreated and treated clayey sandy soil with various percentages of micro-Illite in different curing periods.

The results on the graph in Fig. 8 of axial strain versus axial stress showed that the addition of micro-Illite to untreated clayey sandy soil influenced the mechanical properties of the untreated specimens.

**Figure 9:**
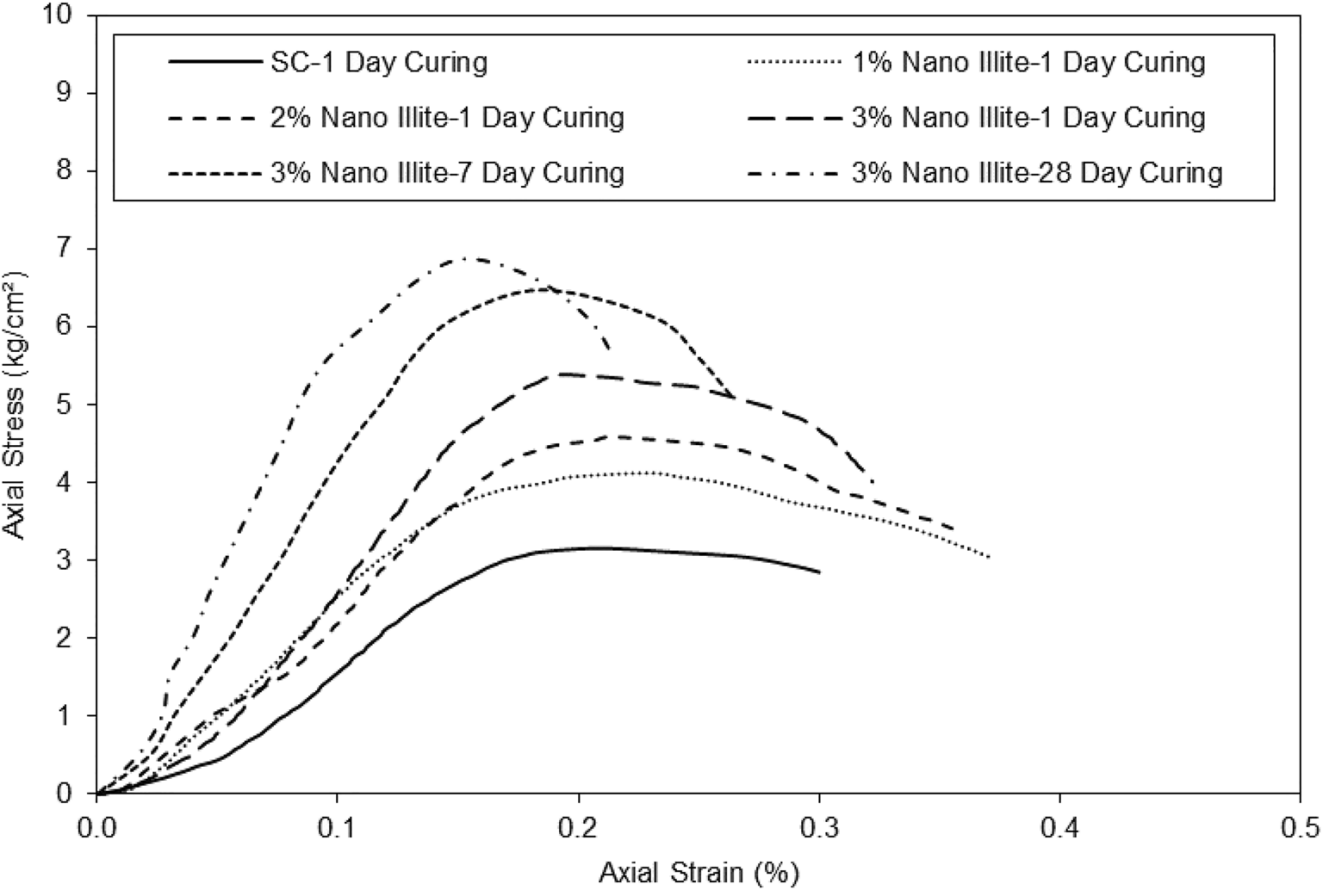
Stress shear strength versus strain for untreated and treated clayey sandy soil with various percentages of micro-Illite in different curing periods.

The results suggest that the addition of micro-Illite can be used to strengthen clayey sandy soils and improve their ability to resist deformation and failure under heavy loads in various curing times. The clayey sand curve at 1d of cure increases gradually until it reaches its maximum with an axial stress of 3 kg/cm^2^ for an axial strain of 0.18%. The treated clayey sandy specimen with the addition of 1% micro-Illite at 1d of maturation reaches its maximum optimum at an axial stress of 2.8 kg/cm^2^ for an axial strain of 0.18%. Adding 1% micro-Illite to the clayey sand specimen decreased the axial strain of the sample. The clayey sand sample with the addition of 2% micro-Illite at 1d of maturation reached its maximum optimum at an axial stress of 3.4 kg/cm^2^ for an axial strain of 0.14%. The clayey sand sample with the addition of 3% micro-Illite at one day of maturation reaches its maximum optimum at an axial stress of 2.9 kg/cm^2^ at an axial strain of 0.12%. Increasing the micro-Illite content in the clayey sandy specimens decreased the axial strain. The clayey sand sample with the addition of 3% micro-Illite at 7d curing times reaches its maximum optimum at an axial stress of 3.3 kg/cm^2^ for an axial strain of 0.14%. The clayey sand sample with the addition of 3% Illite at 28d of maturation reaches its maximum optimum at an axial stress of 3.4 kg/cm^2^ for an axial strain of 0.12%. Adding 3% micro-Illite to the sample increased the axial stress by 0.45 kg/cm^2^ (3kg/cm^2^/3kg/cm^2^) in 7 days curing times. It can be assumed that the addition of 3% micro-Illite to clayey sand stabilizes the axial stress as a function of time. However, it can also be assumed that the axial stress and strain stabilize after 7d. There is not much difference between the curve representing the addition of 3% Illite at 7d of cure and the curve representing the addition of 3% Illite at 28 days of cure. All curves with added micro-Illite look similar. After conducting unconfined compression tests on clayey sandy samples with different concentrations of nano-Illite, the results on the graph in Fig. 9 show a significant reduction in axial strain versus axial stress for samples containing nano-Illite.

Figure 9 shows the axial strain as a function of the axial stress of the clayey sand with the addition of nano-Illite. The curve of the clayey sand at 1d curing increases progressively until it reaches its maximum with an axial stress of 3 kg/cm^2^ for an axial strain of 0.18%. The clayey sand specimen with the addition of 1% nano-Illite content at 1 day curing reaches its maximum optimum at an axial stress of 4.2 kg/cm^2^ for an axial strain of 0.23%. The clayey sand sample with the addition of 2% nano-Illite content at 1d curing reaches its maximum optimum at an axial stress of 4.5kg/cm^2^ for an axial strain of 0.22%. The clayey sand sample with the addition of 3% nano-Illite content at 1 day curing reaches its maximum optimum at an axial stress of 5.2kg/cm^2^ for an axial strain of 0.2%. It can be assumed that the more additive is added the more axial stress and axial strain increase.

**Figure 10:**
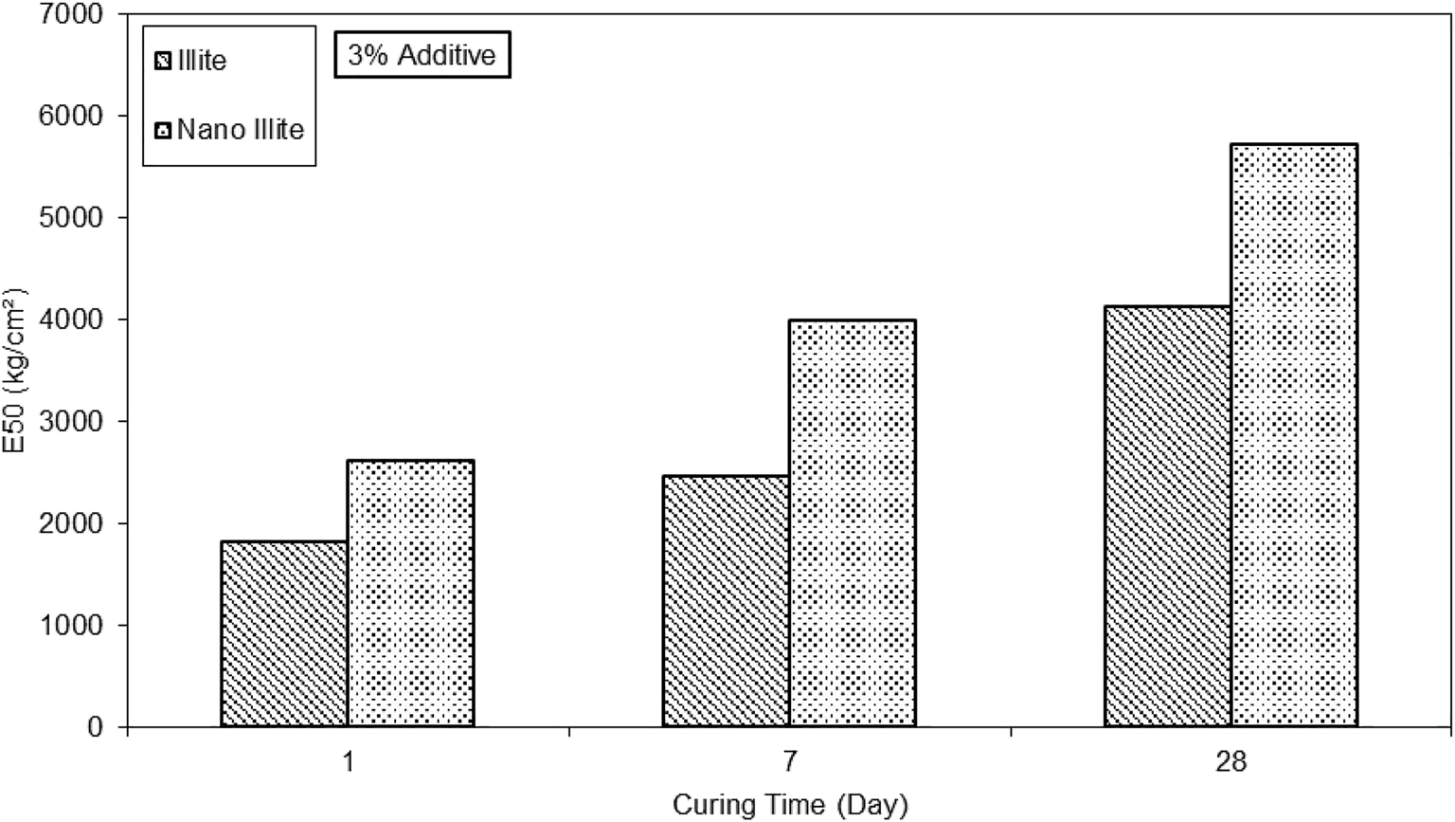
E50 versus various curing times for treated clayey sandy soil with 3% formulations of micro- and nano-Illite.

The clayey sand sample with the addition of 3% nano-Illite content at 7d curing reaches its maximum optimum at an axial stress of 6.5 kg/cm^2^ for an axial strain of 0.20%. The clayey sand sample with the addition of 3% nano-Illite content at 28d curing reaches its maximum optimum at an axial stress of 7 kg/cm^2^ for an axial strain of 0.16%. It can be seen again that at 7d curing, the clayey sand sample with the addition of 3% nano-Illite content stabilizes since at 28 days the curve doesn't show a large deviation. Observations suggest that the addition of nano-Illite improves the resistance to axial deformation of clayey sand by acting as a binder. The results on the graph in bar of Fig. 10 show the days of cures according to the secant modulus at 50% of peak stress of the micro-Illite and the nano-Illite with 3% of additive.

On 1d, the secant modulus at 50% of peak stress (E50) of micro-Illite and nano-Illite are 1950 kg/cm^2^ and 2500 kg/cm^2^ respectively. So, on day 1, the secant modulus at 50% of peak stress of micro-Illite is greater than that of nano-Illite. On 7d, the secant modulus at 50% of peak stress of micro-Illite and nano-Illite are 2300 kg/cm^2^ and 4050 kg/cm^2^ respectively. A reduction in the secant modulus at 50% of peak stress are observed for micro-Illite while that of nano-Illite doubles. On 28d, the E50 of micro-Illite and nano Illite are 4100 kg/cm^2^ and 5800 kg/cm^2^ respectively. A further decrease in the secant modulus at 50% of peak stress is observed for the micro-Illite while that of the nano-Illite continues to increase. A decrease in secant modulus at 50% of peak stress over time is observed for micro-Illite. However, a strong increase in secant modulus at 50% of peak stress over time is observed for nano-Illite. On 1d, the E50 is 2300 kg/cm^2^ and on 28 d, it is 5800 kg/cm^2^. The E50 is 3 times higher on day 28 than on 1d. Observations suggest that nano-Illite with 3% additive has better secant modulus at 50% of peak stress over time, whereas that of micro-Illite degrades.

**Figure 11:**
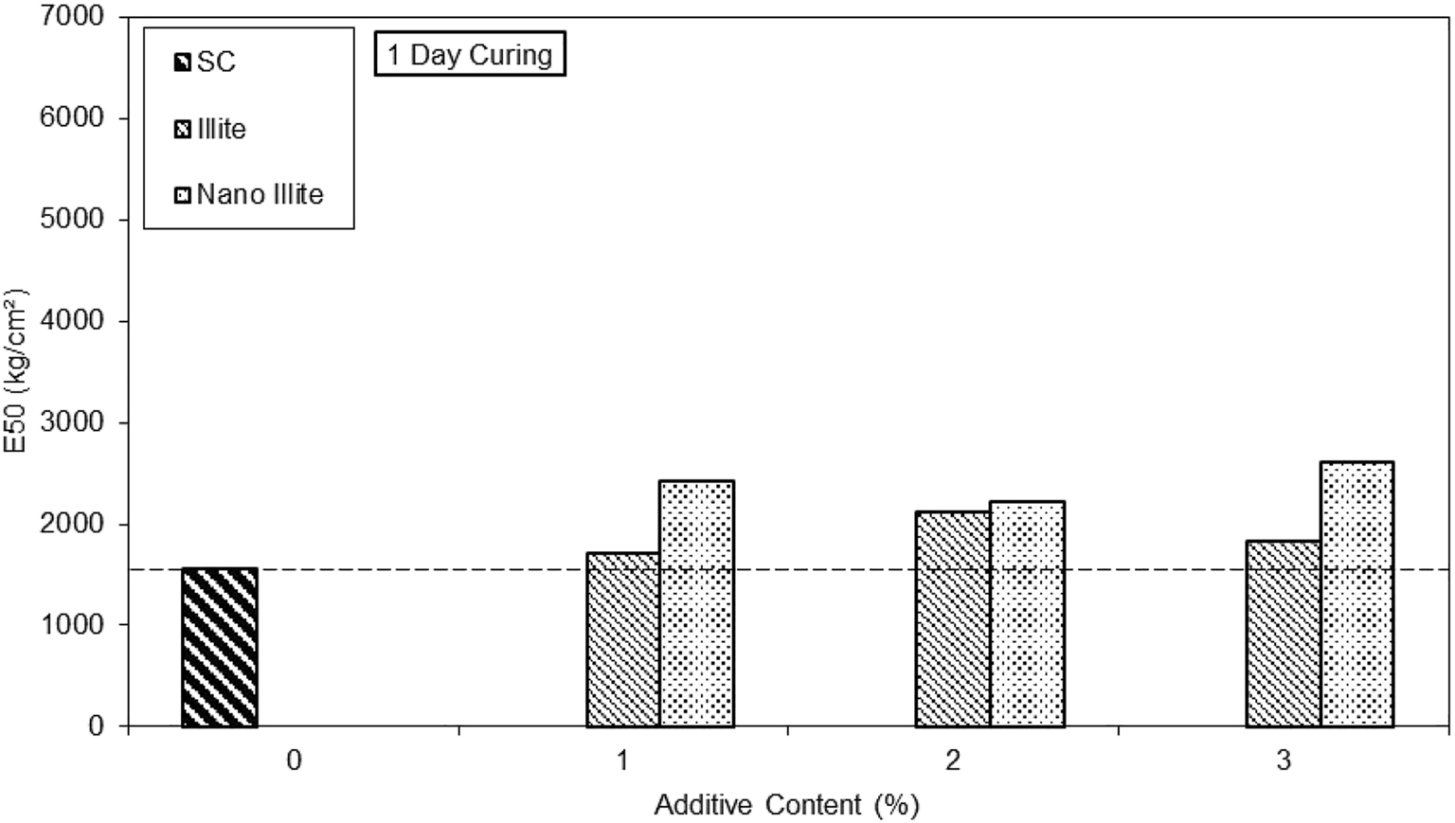
E50 versus untreated and treated clayey sandy soil specimens with various percentages of formulations of micro- and nano-Illite in 1 day curing time.

The results on the bar graph in Fig. 11 show the additive content as a function of the secant modulus at 50% of peak stress of clayey sandy soil, micro-Illite and nano-Illite at 1 day of curing time.

The clay sand without additives achieves a secant modulus at 50% of peak stress of 1650 kg/cm^2^. With the addition of 1% of additives, the micro-Illite and the nano-Illite reach respectively 1700 kg/cm^2^ and 2400 kg/cm^2^ at one day of curing time. With the addition of 2% of additives, the micro-Illite and the nano Illite reach respectively 2100 kg/cm^2^ and 2180 kg/cm^2^ at one day of curing time. With the addition of 3% of additives, the micro-Illite and the nano-Illite reach respectively 1800 kg/cm^2^ and 2500 kg/cm^2^ at one day of curing time.

The secant modulus at 50% of peak stress of micro-Illite and nano-Illite with the addition of an additive is higher than that of untreated clay sandy soil. Observations suggest that nano-Illite with 1% additive has better secant modulus at 50% of peak stress on 1d curing time than that of micro-Illite. However, at 1 d curing time, with 2% and 3% additive content, nano-Illite has a higher secant modulus at 50% of peak stress than that of micro-Illite, which confirms our results in Fig. 11.

### SEM

**Figure 12:**
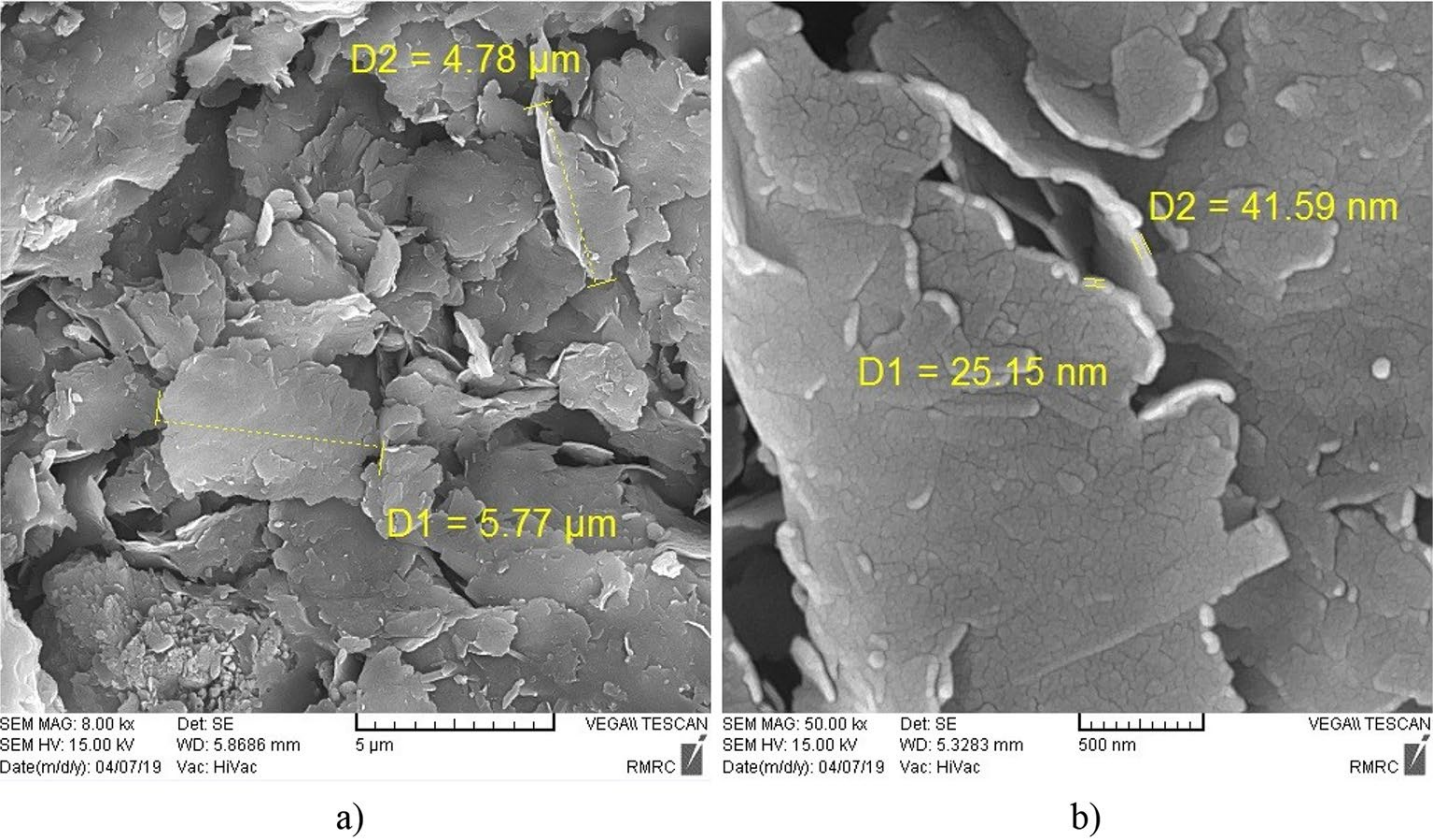
SEM micrographes of: (**a**) treated clayey sandy soil sample with micro-Illite additives, (**b**) untreated clayey sandy soil sample for 1d curing times.

**Figure 13:**
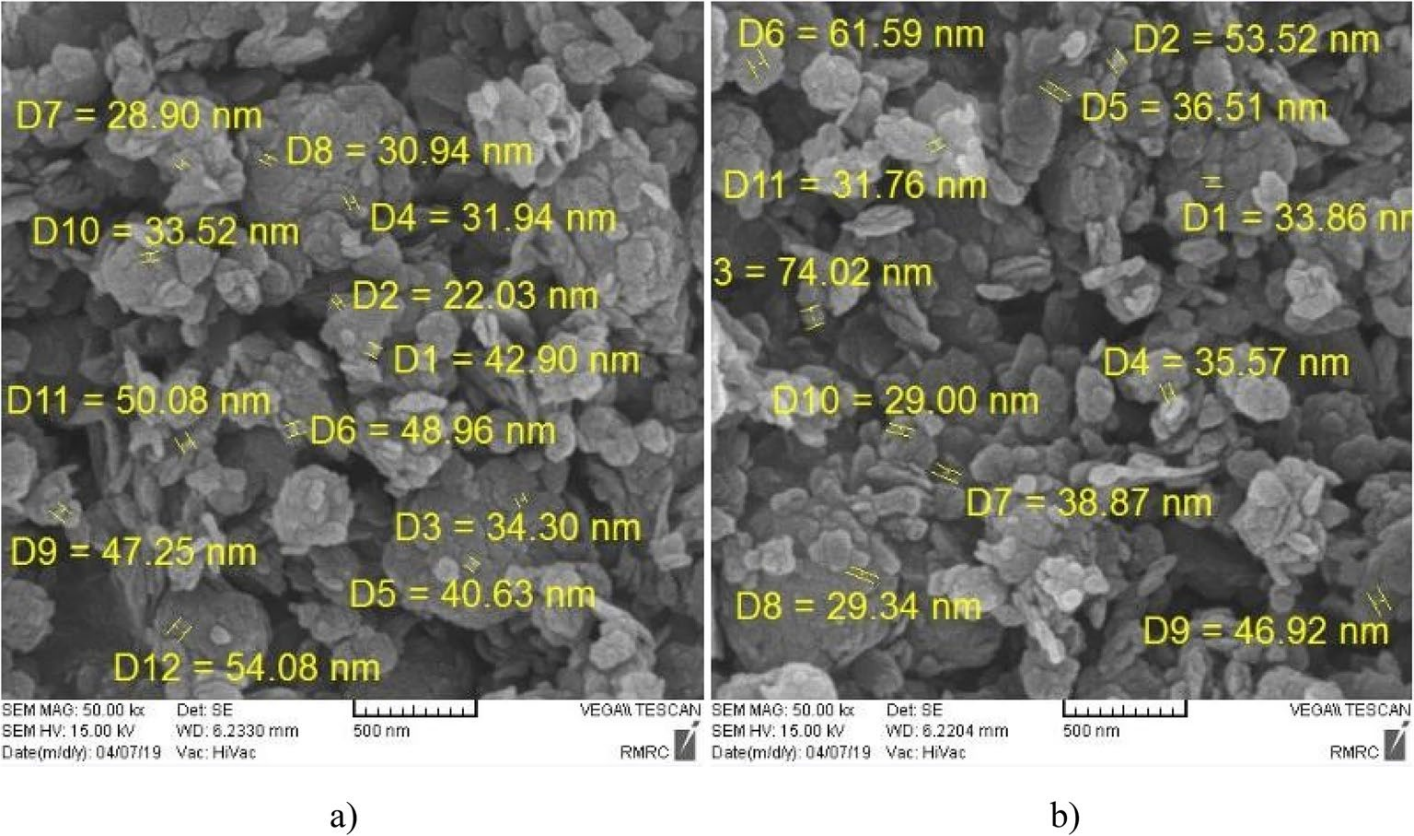
SEM micrographes of: (**a**) treated clayey sandy soil sample with nano-Illite additives for 28d curing time, (**b**) treated clayey sandy soil sample with nano-Illite additives for 7d curing times.

Examining the morphology of clayey sandy specimens with micro- and nano-Illite, Scanning Electron Microscopy (SEM) showed the cementation mechanism that leads to improving soil samples. SEM involves using an electron beam to scan the surface of a sample and produce a two-dimensional image of its surface. Untreated and treated soils are very complex where it is not easy to demonstrate the micro- and nano-particles using SEM although micro- and nano-Illites were observe in various SEM images that shown in Figs. 12 and 13. Various micro-Illites type structures, were seen with micro-size scale in different percentages 0%, 1%, 2%, and 3% in specimens, shown in Fig. 12a. Untreated clayey sand soil sample observed the morphology in Fig. 12b. High-resolution observation showed that micro-Illite particles are arranged in layers and have a sheet-like shape, which could explain their ability to layer on top of each other to form an aggregated particle structure. The particles were also observed with relatively irregular edges and a rough surface with asperities of different sizes. In conclusion, micro-Illite has a sheet shape which could account for its ability to layer and clump together, making it a potential additive for improving clayey sandy soils.

Scanning electron microscope analysis of the nano-Illite particles in Fig. 13 revealed a uniform and relatively small particle aggregate structure. The nano-Illite (NI) additive increased particle aggregation with the gel formation holding the soil particles together that it is cause of higher reactivity of nano-Illite to occur with clayey sandy particles. High-resolution observation showed that the nano-Illite particles have a spherical shape, with a smooth and irregular surface. This spherical shape and smooth surface could explain the dispersion stability of nano-Illite in aqueous solutions. In addition, nano-Illite has higher porosity and permeability than traditional Illite. This could increase its effectiveness as an additive to improve clayey sandy soils.

In order to confirm the nano-Illite additive reach to maximum strength improvement in clayey sand soil specimens including 3% nano-Illite, samples were evaluated with SEM. SEM images provided good observation about the nano-Illite process and change of surface compositions in different clayeye sandy soil samples.

### XRD

**Figure 14:**
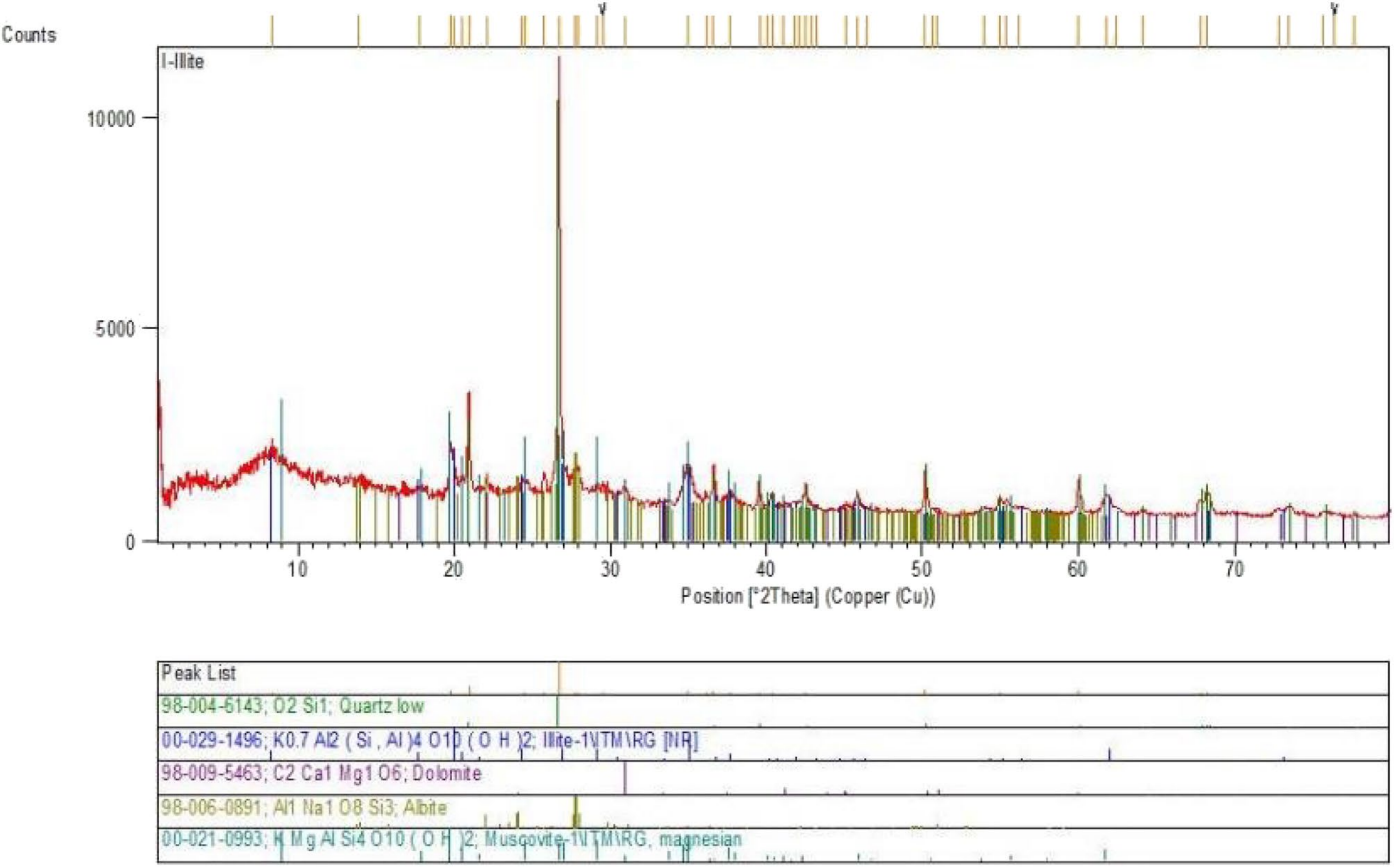
XRD pattern of treated clayey sandy soils with micro-Illite.

X-ray diffraction (XRD) testing is a method used to study the crystal structure of a substance. In order to confirm the nano-Illite additive reach to maximum strength improvement in clayey sand soil specimens including 3% nano-Illite, samples were evaluated with SEM. The results showed that micro-Illite is characterized by a very fine and homogeneous crystalline structure. The diffraction peaks observed on the graph of Fig. 14.

In general, the results of the X-ray diffraction (XRD) test confirm that micro-Illite is indeed a smectite clay and show that the crystal structure of Illite is very fine and homogeneous. This kind of crystal structure is very favorable for geotechnical applications, as it can improve the compressive strength and stability of soft soils. Figure 14 shows the XRD pattern of treated clayey sandy soils with micro-Illite. In conclusion, the results of the X-ray diffraction test for micro-Illite are very promising for geotechnical applications and suggest that micro-Illite can be used effectively to improve the properties of clayey sandy soils.

**Figure 15:**
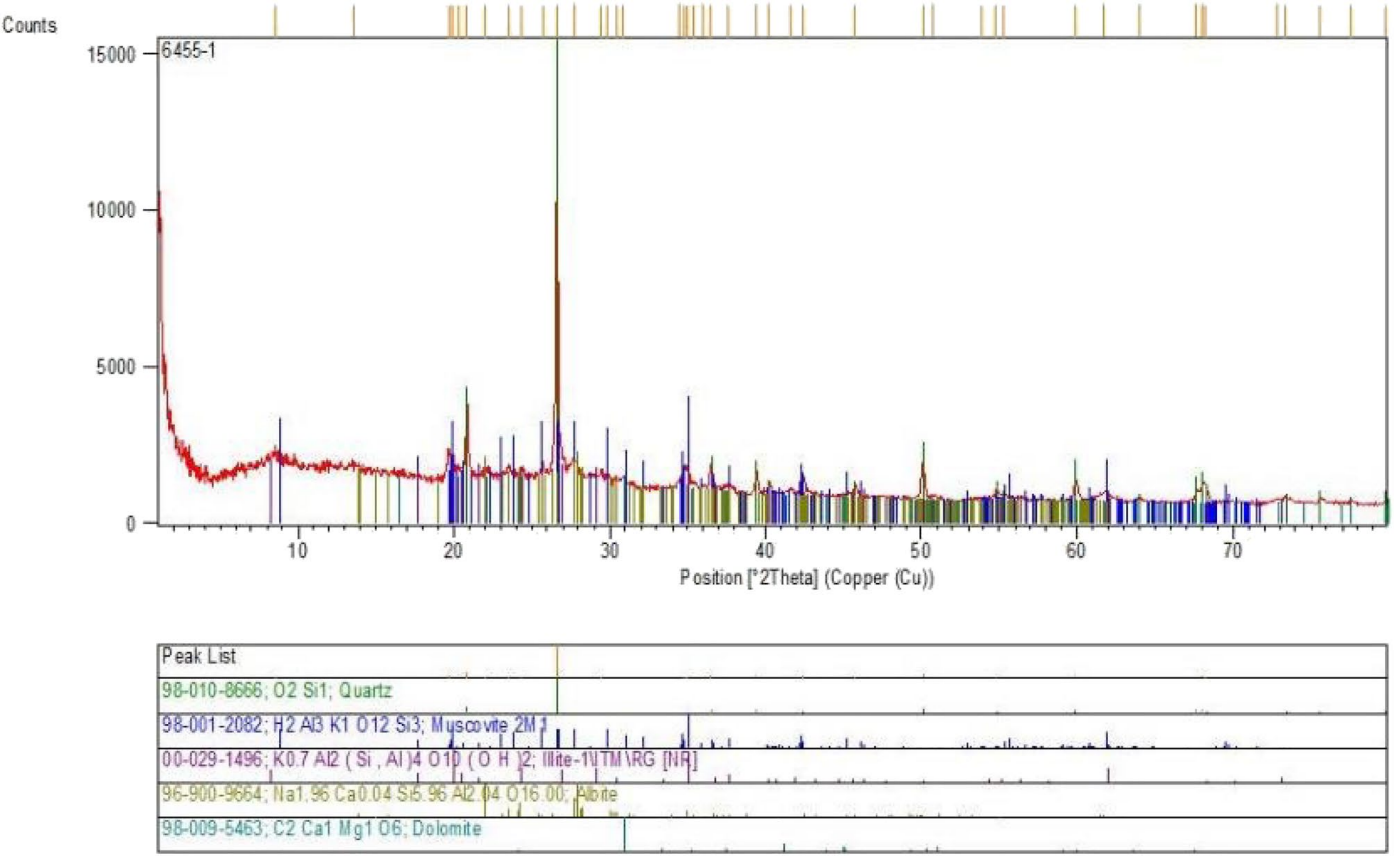
XRD pattern of treated clayey sandy soils with nano-Illite.

Observing the results of the X-ray diffraction test for nano-Illite shows distinct and well-defined peaks at specific diffraction angles. The diffraction peaks on the graph in Fig. 15 correspond to specific distances between the atoms of the nano-Illite, indicating a well-defined and organized crystal structure.

The results of this test show that nano-Illite has a different crystal structure than conventional micro-Illite, which may explain the differences in the properties of these two materials.

In order to confirm the nano-Illite additive reach to maximum strength improvement in clayey sand soil specimens including 3% nano-Illite, samples were evaluated with XRD. In conclusion, the results of the X-ray diffraction test show that nano-Illite has a well-defined crystal structure different from that of conventional micro-Illite, which may explain the differences in their properties. The observations from this test are very important for the improvement of clayey sandy soils, as they show that nano-Illite can be a promising material for this application due to its unique properties.

## Conclusions

This research explores the potential of micro- and nano-Illite as soil stabilizers for improving the geotechnical properties of clayey sandy soils. Illite is a type of clay mineral that can form nano-cementation (NC) in soil, which is a process of enhancing the durability of various building materials. NC is also known as nano soil-improvement (NSI), a technique that has been developed in recent years. The main objective of this study was to compare the performance of micro- and nano-Illite as soil stabilizers and to evaluate their effects on the strength and consistency of clayey sandy soils. Four formulations of micro- and nano-Illite with concentrations of 0, 1, 2, and 3% were separately added to soil samples. The unconfined compressive strength (UCS) and the secant modulus at 50% of peak stress (E50) of the treated samples were measured and compared with the untreated samples. The results showed that nano-Illite significantly improved the UCS and E50 of soil by more than 2.2 times and 1.5 times, respectively, after 7 days of curing. Micro-Illite also improved the UCS and E50 of soil, but to a lesser extent. Atterberg limit tests showed that nano-Illite reduced the liquid limit, plastic limit, and plasticity index of soil and increased its consistency. X-ray fluorescence (XRF), scanning electron microscopy (SEM), and X-ray diffraction (XRD) analyses revealed the micro- and nano-structures of the soil specimens and the performance of Illite as a nano-additive. The results suggest that nano-Illite has a spherical shape, a smooth and uniform surface, and an ability to increase the stability of soil, which make it superior to micro-Illite and traditional Illite as a soil stabilizer. This study demonstrates the effectiveness of nano-Illite in soil improvement as a NSI technique, and its novelty lies in the direct application of micro- and nano-Illite in soil stabilization. This research was done at a laboratory scale, and future work is required to conduct large-scale and field tests.

## Data Availability

The datasets generated during and/or analysed during the current study are available from the corresponding author on reasonable request.
